# Localized PD-1 Blockade in a Mouse Model of Renal Cell Carcinoma

**DOI:** 10.3389/fddev.2022.838458

**Published:** 2022-05-09

**Authors:** Ngoc B. Pham, Nevil Abraham, Ketki Y. Velankar, Nathan R. Schueller, Errol J. Philip, Yasmeen Jaber, Ellen S. Gawalt, Yong Fan, Sumanta K. Pal, Wilson S. Meng

**Affiliations:** 1 Graduate School of Pharmaceutical Sciences, Duquesne University, Pittsburgh, PA, United States,; 2 School of Medicine, University of California, San Francisco, San Francisco, CA, United States,; 3 Department of Medical Oncology and Developmental Therapeutics, City of Hope Comprehensive Cancer Center, Duarte, CA, United States,; 4 Department of Chemistry and Biochemistry, Duquesne University, Pittsburgh, PA, United States,; 5 McGowan Institute for Regenerative Medicine, University of Pittsburgh, Pittsburgh, PA, United States,; 6 Institute of Cellular Therapeutics, Allegheny-Singer Research Institute, Pittsburgh, PA, United States,; 7 Department of Biological Sciences, Carnegie Mellon University, Pittsburgh, PA, United States

**Keywords:** hydrogel, self-assembling peptides, EAK16-II, RENCA, immune checkpoint blockade, peritumoral delivery

## Abstract

Herein we report the impact of localized delivery of an anti-mouse PD-1-specific monoclonal antibody (aPD1) on Renca tumors in the resulting T cell responses and changes in broader immune gene expression profiles. Renca is a BALB/c mice syngeneic tumor that has been used to model human renal cell carcinoma In this study, T cell subsets were examined in tumors and draining lymph nodes of mice treated with localized PD-1 with and without the addition of adenosine deaminase (ADA), an enzyme that catabolizes adenosine (ADO), identified as an immune checkpoint in several types of human cancers. The biologics, aPD1, or aPD1 with adenosine deaminase (aPD1/ADA), were formulated with the self-assembling peptides Z15_EAK to enhance retention near the tumor inoculation site. We found that both aPD1 and aPD1/ADA skewed the local immune milieu towards an immune stimulatory phenotype by reducing Tregs, increasing CD8 T cell infiltration, and upregulating IFNɣ. Analysis of tumor specimens using bulk RNA-Seq confirmed the impact of the localized aPD1 treatment and revealed differential gene expressions elicited by the loco-regional treatment. The effects of ADA and Z15_EAK were limited to tumor growth delay and lymph node enlargement. These results support the notion of expanding the use of locoregional PD-1 blockade in solid tumors.

## INTRODUCTION

Although outcomes for metastatic renal cell carcinoma (RCC) have improved drastically, for most patients, it remains a lethal disease. The immunogenic nature of RCC raised the prospect that immune checkpoint inhibitors (ICI), including anti-PD-1 antibodies (aPD1), could improve outcomes of those with advanced disease (Weinstock and McDermott, 2015; Rini et al., 2019). Indeed, the CheckMate-025 trial shown an overall response rate (ORR) of 25% to nivolumab (a marketed aPD1) in patients with metastatic clear cell RCC (Motzer et al., 2015). Combination therapy with dual ICI therapy, blocking both PD1 and CTLA4 with the combination of nivolumab with ipilimumab, raised ORR to nearly 50%, with the potential for long-term durable responses in a subset of patients (Motzer et al., 2019). Combinations of targeted therapy with ICI now represent the mainstay of treatment, with ORR reaching 55–71% (Rini et al., 2019; Choueiri et al., 2021; Motzer et al., 2021). While antibodies directed against PD-1 are approved as first line agents, a significant proportion of patients still fail to respond to such therapy (Rini et al., 2019).

The concept of loco-regional drug delivery has gained substantial interest in the immuno-oncology (IO) domain (Marabelle et al., 2017). Directly injecting drugs into tumor lesions enables higher drug concentrations in tumors and draining lymph nodes than can be achieved via systemic infusion, while at the same time reducing the risk of immune-related adverse events. In a pivotal trial evaluating nivolumab with ipilimumab for metastatic RCC, the rate of grade 3/4 toxicity was 46% (Flippot et al., 2018). In contrast, local injection of anti-CTLA-4 antibodies in mouse models was shown to increase effector T cells and reduce Tregs in distant tumors, with limited systemic toxicities (Fransen et al., 2013). Intratumoral (i.t.) injections of ipilimumab alone or in combination with cytokines, TLR ligands, radiotherapies or cell therapies have been investigated in the clinical trial setting (NCT03707808, NCT02812524, NCT02977156, NCT01672450, NCT03233152, NCT02254772, NCT04270864, NCT02857569). Importantly, anticancer agents injected i. t. are not necessarily confined to tumors, due to solid tumors being perfused with leaky blood capillaries and irregular lymphatic vessels. Because of the high interstitial fluid pressure, i. t. injected drugs, without a retention mechanism, might also be subject to rapid elimination and not achieve their intended anti-tumor effects.

Extending the retention of IO antibodies in the TME should enhance efficacy (Fransen et al., 2011; Ishihara et al., 2017). The phenotype and infiltration of T cells depends on the changing concentrations of tumor-specific antigens, the phenotypic plasticity of antigen presenting cells, and the flux of cytokines and metabolites (Lanitis et al., 2017). In such a dynamic setting, it is challenging to design optimal i. t. dosing regimens for monoclonal antibodies targeting immune markers. Transient drug concentrations from a bolus injection may fail to coincide with the expression of the immune targets. Furthermore, the location and fibrotic nature of many tumors limits the potential for frequent i. t. injections. The need for local sustained release of IO drugs motivated us to develop an injectable hydrogel for delivering aPD1 antibodies to the tumor region. Peritumoral administration of anti-CD40 antibodies formulated in microparticles has been reported to release antibodies in a sustained manner and enhanced tumor-specific CD8 T cells (Fransen et al., 2011).

Adenosine (ADO) is an immune checkpoint metabolite that suppresses multiple cellular and molecular mechanisms responsible for antitumor immunity (Leone and Emens, 2018). Several ongoing clinical trials are being conducted to investigate the effects of targeting the ADO pathway in RCC (e.g., NCT03454451, NCT04306900, NCT04262375, NCT02655822, NCT03207867, NCT04306900, NCT03549000). Extracellular ADO is generated from extracellular ATP, a pro-inflammatory mediator that accumlates up to hundreds of micromolar concentrations in the TME (Pellegatti et al., 2008). Extracellular ADO has a very short half-life (less than 10 s in whole blood) but accumulates in the TME (Klabunde, 1983; Ohta et al., 2006). *In vivo* microdialysis methods revealed extracellular ADO concentrations ranging from 100 nM to 2.5 μM in murine solid tumors (Blay et al., 1997; Willingham et al., 2018). ADO can be degraded on demand using adenosine deaminase (ADA), which irreversibly deaminates adenosine to inosine (Fischer et al., 1976). ADA has been used clinically for decades in enzyme replacement therapies.

Because ADO acts through autocrine and paracrine feedback loops in the TME, it is imperative that aPD1 and ADA are concentrated in the TME at the same time for extended durations. A gel-formulated aPD1 IgG and ADA would enable sustained local concentrations of these agents that cannot be achieved via systemic infusions. In this study, we used peptidic fibrils assembled from Z15_EAK, a 15-amino acid Fc-binding peptide fused with a fibrillar self-assembling peptide (SAP) (AEAEAKAKAEAEAKAK, or “EAK”) we developed (Pham et al., 2019). The bi-functional peptide Z15_EAK did not induce acute inflammation or toxicities in mice and could be injected using a conventional 25 gauge needle syringe to render multivalent Fc-binding sites *in vivo*. We tested this strategy in BALB/c mice bearing the syngeneic Renca tumors that have been used to model human RCC (Murphy and Hrushesky, 1973). We found that aPD1/ADA gel increased CD8^+^ T cells and IFNɣ in tumors and decreased CD4+FoxP3+ regulatory T cells (Tregs) in tumor draining lymph nodes. Bulk RNA-Seq analysis revealed differential upregulation and downregulation of immune and metabolic genes as a function of the treatments. Taken together, these data indicated that the Renca TME could be modulated by local delivery of aPD1 to promote a Th1-type T cell response.

## MATERIALS AND METHODS

### Cell Line

Renca (ATCC^®^ CRL-2947^™^) was purchased and sub-cultured at 37°C, 5% CO_2_. The cell line was maintained in a RPMI-1640 complete medium containing 10% FBS, sodium pyruvate, non-essential amino acid, l-glutamine, and penicillin streptomycin. The complete medium used in *vitro* culture with lymphocytes was supplemented with 50 μM 2-mercaptoethanol. Trypsin-EDTA solution was utilized to detach Renca cells following ATCC^®^’s cell passage protocol. Renca cells from passage 3 to 14 were used in the present study.

### Mouse RCC Model

Eight-to twelve-week-old female BALB/c mice were purchased from Hilltop laboratory Animals (Scottdale, PA) and housed in the Duquesne University Animal Care Facility. Animals were handled in accordance with protocols approved by the Duquesne University Institutional Animal Care and Use Committee. Hairs on the upper right abdomen were shaved prior to cell inoculation and injections. A P0 BALB/c mouse was inoculated with 2 × 10^6^ Renca cells that were in the proliferation state. The primary tumor was collected 15–17 days after inoculation and processed for further *in vivo* inoculation (see Tumor Dissociation method in the Supplementary Data Sheet S1). Processed tumor cells (1–2 × 10^6^) were suspended in HBSS and inoculated subcutaneously (s.c.) to an average set of 10 BALB/c mice.

The first dose of treatment or control was injected peritumorally (s.c.) on day 3 after inoculation, following by two more doses on day 6 and day 10. Full treatment components included 0.2 mg aPD1 (10 mg/kg equivalent), 0.2 mg ADA (5 mg/ml), and 0.117 mg Z15_EAK (5 mg/ml). Tumors (width and length) were measured every 2–4 days with an electronic caliper. Tumors and inguinal lymph node were harvested 2 days after the last dose. Specimens were weighed immediately after collection (Metter Toledo ME54E balance). End-point tumors were placed immediately in RNAlater solution and stored at −20°C until PCR analysis. Animals with tumors showing signs of infection were euthanized and excluded from the analysis.

### Draining Lymph Nodes Cell Isolation

The inguinal lymph node is responsible for draining the site of injection. dLN were stored in RPMI complete media, on ice, and weighed immediately upon collection. dLN were crushed against a 40 μm strainer using a sterile syringe plunger. The cell lysate was then centrifuged and resuspended for cell counting. The number of cells were equilibrated across the samples for flow cytometry staining and *ex vivo* cell culture plating.

### Flow Cytometry

dLN cell staining was performed in low-retention microcentrifuge tubes and with reagents provided in the mouse Regulatory T Cell Staining Kit (eBioscience^™^). Anti-mouse CD16/CD32 was first used to block non-specific Fc binding. Following procedure described in the kit protocol, the cells were stained with 0.125 μg anti-mouse CD4 FITC (RM-5) and 0.5 μg anti-mouse/rat FoxP3 PE (FJK-16s). Samples in the study were analyzed with the Attune NxT flow cytometer (Thermo Fisher). On average, 10^5^ events were run, with gates to exclude debris and doublet cells. AbC^™^ Total Antibody Compensation Bead Kit (Invitrogen) was employed to adjust color compensation.

### *Ex vivo* T Cell Cultures and ELISA

Renca and dLN cells were seeded at the ratio of 1:10 in a 96-well plate with a final volume of 250 μL. Each well was supplemented with 20 ng/ml recombinant mouse IL-2 (R&D systems) to stimulate lymphocyte proliferation. After an overnight incubation at 37°C, 5% CO_2_, the cell suspension was centrifuged, and the supernatant was analyzed with an IFNɣ mouse uncoated ELISA kit (Invitrogen). Each cell culture sample was tested as triplicates in the ELISA assay and detected with the TECAN infinite M1000 microplate reader (Männedorf, Switzerland). The concentration interpolation was obtained from an 8-point or 5-point standard curve (15–2,000 pg/ml).

### RT-qPCR

RNA was isolated using a TRIzol Extraction kit. Whole tumors were homogenized in TRIzol solution using a Tumor Homogenization Kit (GentleMACS, Miltenyi Biotech). RNA extraction was performed according to manufacturer protocol. The RNA concentration and quality were analyzed using an RNA nano 6000 kit (Agilent Technologies). Each RNA sample was run in triplicate, and the results from the Agilent 2100 Bioanalyzer were averaged to obtain a final concentration and RNA Integrity Number (RIN). Samples with a RIN >8 were considered to have good quality RNA. 1 μg of RNA was reverse transcribed using a cDNA Synthesis Kit (SuperScript VILO IV Master Mix, ThermoFisher Scientific). The resultant cDNA was diluted to a final concentration of 5 ng/uL. Samples were analyzed on the Mx3000P Real Time Cycler, with a Taqman Real-Time PCR Master Mix (Applied Biosystems #4304437) and inventoried TaqMan probes from ThermoFisher. 5 μL (25 ng) of each cDNA sample were run singleplex for 100 cycles. ACTB was used as a normalizing gene.

### RNA-Sequencing and Bioinformatics

Extracted RNA were quantitated using the Qubit^™^ RNA BR Assay Kit (Thermo Fisher Scientific) followed by an RNA quality check using the Fragment Analyzer (AATI). Sequencing was performed using NovaSeq 6000 platform (Illumina) to an average of 40M 101PE reads, on the NovaSeq SP-200 flowcell. Gene counts were run through edgeR’s *calcNormFactors* function, specifying the Trimmed Mean of M-values (TMM) algorithm as a means of normalization. Differential gene expression (DGE) analysis was performed with responder status, treatment, cohort, and sequencing batch listed as covariates in a generalized linear model evaluated by a quasi-likelihood F-test in edgeR. *p*-Values were adjusted using the Benjamini–Hochberg procedure to control false discovery rate (FDR). Genes were considered differentially expressed if they met the following criteria:|log_2_| > 1 and adjusted *p*-value < 0.05. More details on the processing of raw data and analysis methodology can be found in the Supplementary Data Sheet S1.

### Statistical Analysis

Statistical analysis was performed in GraphPad Prism 8.0. Data plots shown represent mean and standard mean error. Nonparametric, unpaired *t*-test was conducted for comparison between two groups. Experiments with three tested groups were analyzed using an ordinary one-way ANOVA followed by Dunnett’s multiple comparison tests. Significant values were computed based on two-tailed assumption and marked with asterisks and represented the following: nonsignificant (ns) *p* ≥ 0.05, *p < 0.05, **p < 0.01, ***p < 0.001, ****p < 0.0001. A total of 60 BALB/c mice were involved in the analysis. Mice failed to develop palpable tumors after inoculation within the time frame of the experiment were excluded.

### Hypothesis and Treatment Planning

We hypothesized that locoregional delivery of aPD1 would lead to the induction of a Th1 T cell response in Renca tumors and in tumor draining lymph nodes (dLN). Hydrogels have been used previously to concentrate cytokines and antibodies in tumors to enhance antitumor efficacy (Lv et al., 2017; Chao et al., 2020). In our prior work, we shown that IgG tethered with the SAP EAK could accumulate in epithelial tumors for at least 7 days (Wen et al., 2013). Repeated injections of Z15_EAK and IgG did not elicit acute inflammation or toxicities in mice (Pham et al., 2019). Further, perfusion of IgG complexed with Z15_EAK over Renca cells *in vitro* led to significant accumulation of the antibody on the monolayer (Supplementary Figure S1).

**FIGURE 1 |:**
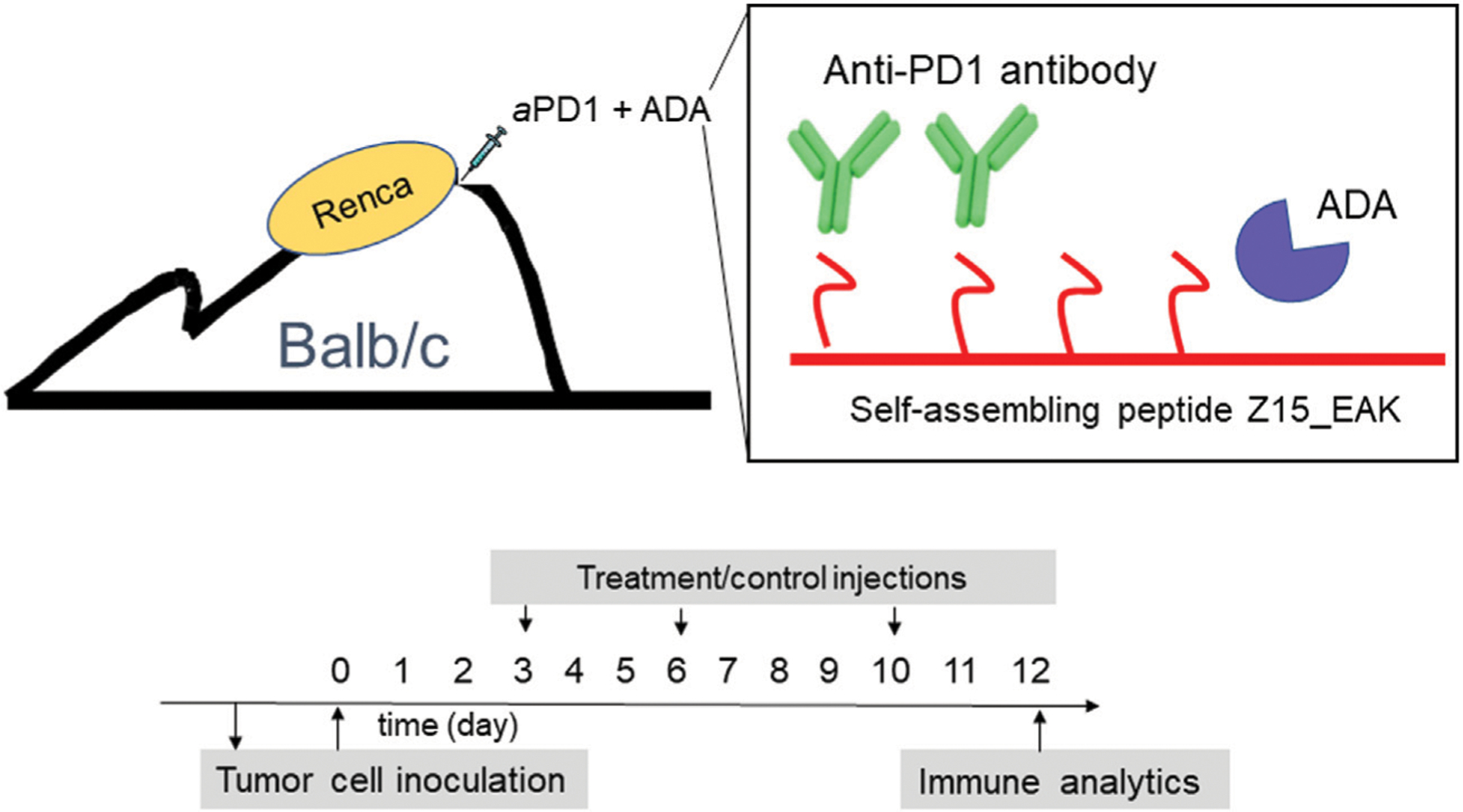
Schematic representations of aPD1/ADA formulated in Z15_EAK gel and dosing strategy. *In vitro* cultivated Renca cells (2 × 10^6) was first injected into the subcutaneous space on the dorsum in a BALB/c mouse to establish an *in vivo* tumor passage. The established tumor was collected after 2 weeks and processed into a single cell suspension using a GentleMACS Tumor Dissociation reagent kit and a GentleMACS Dissociator. *In vivo*-passaged cells were then subcutaneously inoculated into the dorsum of another set of BALB/c mice. Treatments were administered subcutaneously in the tumoral region starting on day 3 post-inoculation. A total of three injections were given two and 3 days apart. Mice were sacrified 2 days after the last dose for *ex vivo* analyses.

The effects of aPD1 antibody (IgG1) and ADA admixed with Z15_EAK (aPD1/ADA gel) on Renca tumors were investigated in Balb/c mice (Figure 1). The tumors used in the experiments were passaged *in vivo* such that each inoculum contained a single cell suspension recovered from a Renca tumor. The *in vivo* passage served to increase tumorigenic properties (Thirunarayanan et al., 2007; Lacoste et al., 2017). Each mouse was injected subcutaneously with 1.5–2 × 10^6^ *in vivo* passaged Renca cells and aPD1/ADA gel was injected subcutaneously adjacent to the inoculation site. The rationale for injecting the gel near the inoculating site (“peritumoral”) is that aPD1 and ADA would slowly diffuse into the tumors and target T cells trafficking to and from dLN (Meng et al., 2020). Altogether, six cohorts of Balb/c mice were inoculated and treated to allow comparison between treatments: aPD1/ADA gel against saline, aPD1/ADA gel against aPD1 gel, and aPD1/ADA gel against aPD1/ADA formulated in saline (aPD1/ADA) (Supplementary Table S1). Each cohort of mice included two to three groups for practical reasons; all the mice in a given cohort received the same cells processed from the same *in vivo* passaged tumor to minimize heterogeneity. For this reason, we believe it is more valid to compare the treatment groups within cohorts. The outcomes of the tumor and lymph nodes analyses were used to stratified into “immune-stimulatory” (IST) or “immune-suppressed” (ISU) as described in the Supplementary Material.

## RESULTS

### Impact of aPD1/ADA Gel on Tumor Growth *in Vivo*

**FIGURE 2 |:**
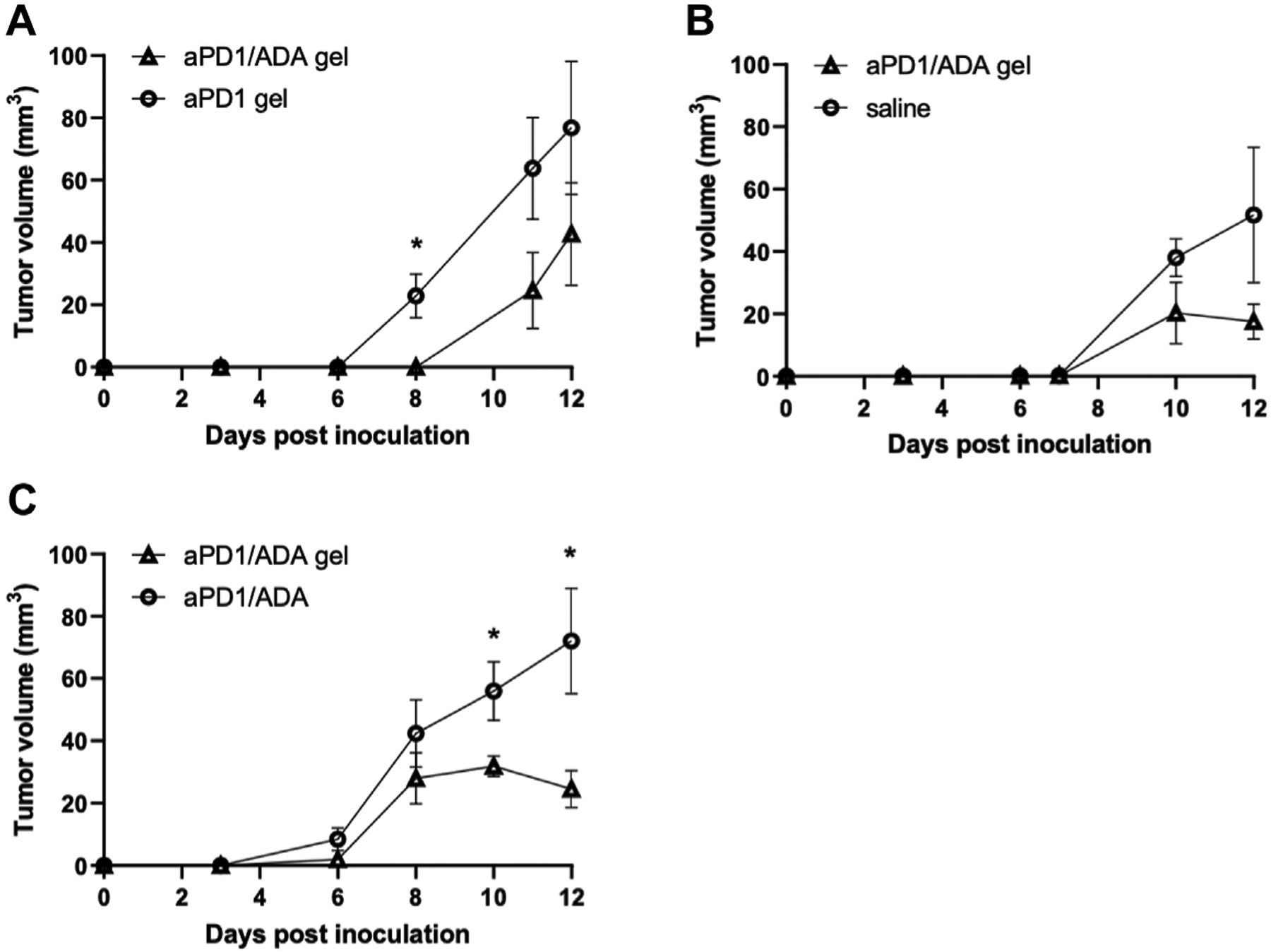
Tumor growth profiles in independent cohorts of mice. Experiments comparing **(A)** aPD1/ADA gel vs. aPD1 gel (B7; *n* = 5), **(B)** aPD1/ADA gel vs. saline (B3; *n* = 5), and **(C)** aPD1/ADA gel vs. aPD1/ADA in saline (B8; *n* = 5). Tumor volumes were calculated using the equation 0.52*(largest dimension*smallest dimension^2^) (Yu et al., 2018). In analysis of early time points, prior to the dimensions could be accurately measured using caliper, very small palpable tumors were assigned 0.5 mm*0.5 mm while small palpable ones 1 mm*1 mm. The unpaired *t*-test was used to determine the significance of difference in volumes at each time point (α = 0.05).

The formulations were evaluated first for their ability to alter tumor growth kinetics. Tumor size was monitored in mice inoculated with Renca cells isolated from *in vivo* passage. Beginning on day three after tumor inoculation, gels containing 0.2 mg of aPD1 and 0.2 mg of ADA (aPD1/ADA gel) were injected subcutaneously around the tumor inoculation site for three doses, with each given 3 days apart (Figure 1). The repeated injections did not induce overt, acute toxicities, and the mice maintained their body weights throughout the experimental periods (Supplementary Figure S2). Given that mice were inoculated with cells harvested from different *in vivo* passaged tumor cells, experiments were conducted in cohorts of mice inoculated with cells isolated form the same passaged tumors in which consistent engraftment could be assumed; each cohort contained mice divided into two to three treatment groups (*n* = 5 mice each) and independently analyzed. In the experiment designated B7, aPD1/ADA gel-treated mice exhibited close to a 2-day delay in emergent of palpable tumors compared to aPD1 gel-treated animals (Figure 2A). In experiment B3, tumors treated with aPD1/ADA gel were smaller from day 6 and onward compared to those treated with saline (Figure 2B). In experiment B8 where mice received either aPD1/ADA gel or aPD1/ADA formulated in saline, the tumors in the former group were significantly smaller toward the end of the monitoring period (Figure 2C). Collectively, these results indicate that ADA and Z15_EAK augmented aPD1 in delaying the growth of Renca tumors.

### aPD1/ADA Gel Enhances Immune Reactivity in Tumors

**FIGURE 3 |:**
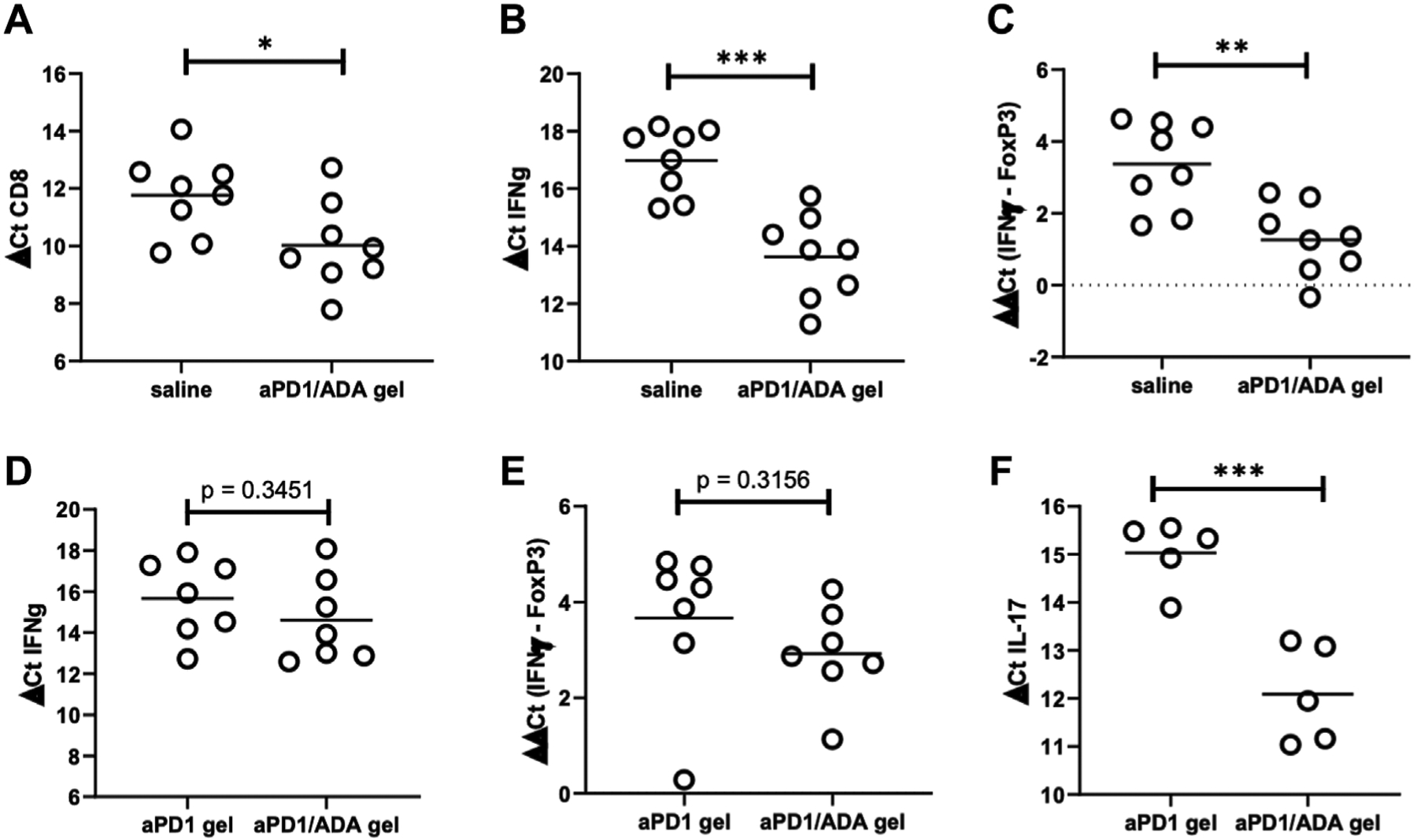
Expressions of CD8, IFNg, and FoxP3 in tumors recovered from two cohorts (B3 and B9) comparing aPD1/ADA gel and saline injections (a third cohort had poor RNA quality (RIN <7) and therefore excluded). RT-qPCR analyses were performed for **(A)** CD8a, **(B)** IFNɣ, and **(C)** IFNɣ relative to FoxP3 (ddCt IFNɣ-FoxP3); lower dCt indicates higher expression; Expressions of the same genes from two other cohorts (B5 and B7) comparing aPD1/ADA gel and aPD1 gel-treated tumors resulted in insignificant differences in **(D)** IFNɣ and **(E)** IFNɣ relative to FoxP3, but significant difference in **(F)** IL-17. RNA were extracted from tumors processed into single cell suspensions using Miltenyi dissociation kits in a GentleMACS. Expressions of the genes were probed with TaqMan primers and normalized to actin. Purities of the RNA were determined using Agilent Nano RNA chip. Significance was determined using unpaired two-tailed *t*-test with *p < 0.05, **p < 0.01, ***p < 0.001, ****p < 0.0001.

The nature of T cells infiltrated into the tumors were characterized using qRT-qPCR. Higher expressions of CD8α and IFNɣ were observed in tumors treated with aPD1/ADA gel compared to those treated with saline among the 16 tumors collected from experiments B3 and B9 (Figures 3A,B). The relative expression of IFNɣ to FoxP3 increased significantly (Figure 3C), but no significant difference was observed in the expression of IL-17A or IL-12a (Supplementary Figure S3A; RNA quality in the third cohort B6, which included aPD1/ADA gel vs. saline treated mice, was exceptionally low therefore the samples were not included in the analysis). Contributions of ADA in the formulation were examined in three cohorts of mice treated with either aPD1/ADA gel or aPD1 gel. In two of the three cohorts, aPD1/ADA gel-treated mice exhibited a trend of increased in IFNɣ expression compared to aPD1 gel (Figure 3D) and a greater shift from FoxP3 to IFNɣ was observed (Figure 3E). In the third cohort, no effect was seen with the addition of ADA (Supplementary Figure S3B). In cohort B7, ADA significantly enhanced the expression of IL-17A (Figure 3F). No difference in CD8α expression was observed between treatment and controls in cohorts treated with aPD1/ADA gel, aPD1 gel, or aPD1/ADA (Supplementary Figures S3C,D). The results suggest potential immune-activating functions of ADA, but additional studies are necessary to establish decisive impact.

### aDP1/ADA Gel Modulates T Cells in dLN

**FIGURE 4 |:**
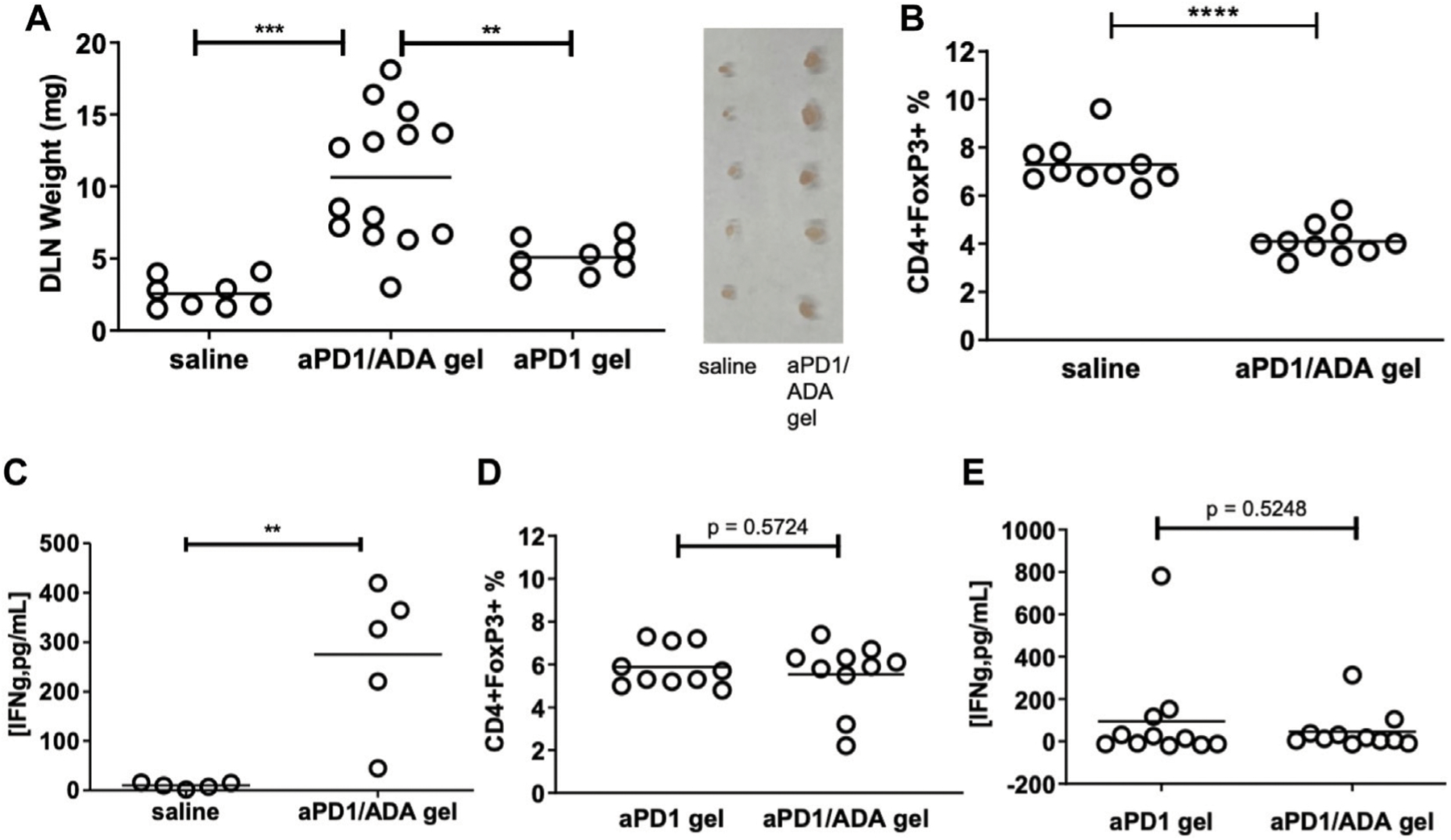
Impact of multiplexed aPD1 and ADA on T cells in tumors and dLN. aPD1/ADA gels were injected into the peri-tumoral region around Renca tumors established subcutaneously in BALB/c mice; **(A)** draining lymph nodes (dLN) isolated 12 days after tumor inoculation and weighted on the same day; insert shows lymph nodes (side-by-side) isolated from mice treated with aPD1/ADA gel or saline; **(B)** Flow cytometric analyses of CD4+FoxP3+ Tregs in dLN collected from mice treated with three doses of saline or aPD1/ADA gel (unpaired two-tailed *t*-test *p* < 0.0001); **(C)** Production of IFNɣ from cultured cells derived from dLN in mice treated with aPD1/ADA gel or saline (*p* < 0.01). **(D)** CD4+FoxP3+ frequencies in dLN recovered from mice treated with aPD1 gel or aPD1/ADA gel. **(E)** IFNɣ released in dLN-derived cells cultured from specimens isolated from mice treated with aPD1 gel or aPD1/ADA gel *p < 0.05, **p < 0.01, ***p < 0.001, ****p < 0.0001.

We next analyzed the T cell subsets developed in the tumor draining lymph nodes in response to the formulations. Inguinal dLN recovered from mice received aPD1/ADA gel weighed five times more than the mice received saline, suggesting an expansion of immune cells (Figure 4A). In delineating the T cell subsets in these dLN, we found lower frequencies of Tregs in the nodes treated with aPD1/ADA gel compared to those received saline (Figure 4B), suggesting a reversal of immune suppression. In cohort B6, higher levels of IFNɣ were detected in *ex vivo* cultures of dLN isolated from aPD1/ADA gel-treated mice than those in mice received saline (Figure 4C). The results suggest that peritumoral injection of aPD1/ADA gel skewed the T cell response in the dLN towards a tumor-rejecting phenotype.

We also compared the effects of aPD1/ADA gel and aPD1 gel on dLN T cells. dLN from mice treated with aPD1/ADA gel were larger than those received aPD1 gel (Figure 4A), indicating that ADA altered the local immune milieu. Both treatments generated approximately the same levels of Tregs and INFɣ in *ex vivo* cultured dLN (Figures 4D,E). The impact of formulating aPD1 and ADA with Z15_EAK on T cell response in the dLN was inconclusive; it could be that peak concentrations in dLN rather than overall exposure of aPD1 at the dose given (0.2 mg) is the decisive factor, and that the depot effect enhances long-term antitumor immunity, which was not measured in the current study. Another possibility is that a stronger gel than Z15_EAK was necessary to enhance retention. This can be accomplished by intermixing Z15_EAK with EAK using a co-assembly strategy (Wen et al., 2014). Nevertheless, while the intended effects of ADA and Z15_EAK on the T cell response were not detected, the enzyme and the gelation biomaterials did delay tumor growth. Taken together, the T cell subsets developed in tumors and dLN and the observed tumor growth point to at least two polarized immune responses, referred in the following narrative as “immune-stimulatory” (IST) and “immune-suppressed” (ISU).

### Differential Gene Expressions in aPD1/ADA Gel-Altered Tumors

RNA-seq was used to delineate the transcriptomic features differentiating tumors responded to aPD1/ADA gel and those did not, and tumors which received the formulation versus saline. Tumors were classified as IST or ISU based on the abundance of CD4+FoxP3+ Tregs, CD8^+^ T cells, and IFNɣ production, as classification metrics, regardless of treatment (Supplementary Figure S4). The RNA-Seq analyses, reported here in dendrograms and plots, revealed unique gene expression patterns from which responding tumors could be defined based on gene expression patterns. The purpose of the analysis was therefore to validate the internal consistencies of the classifications and explore unique signatures of localized aPD1 therapies in the Renca model.

#### Tumor Heterogeneity

**FIGURE 5 |:**
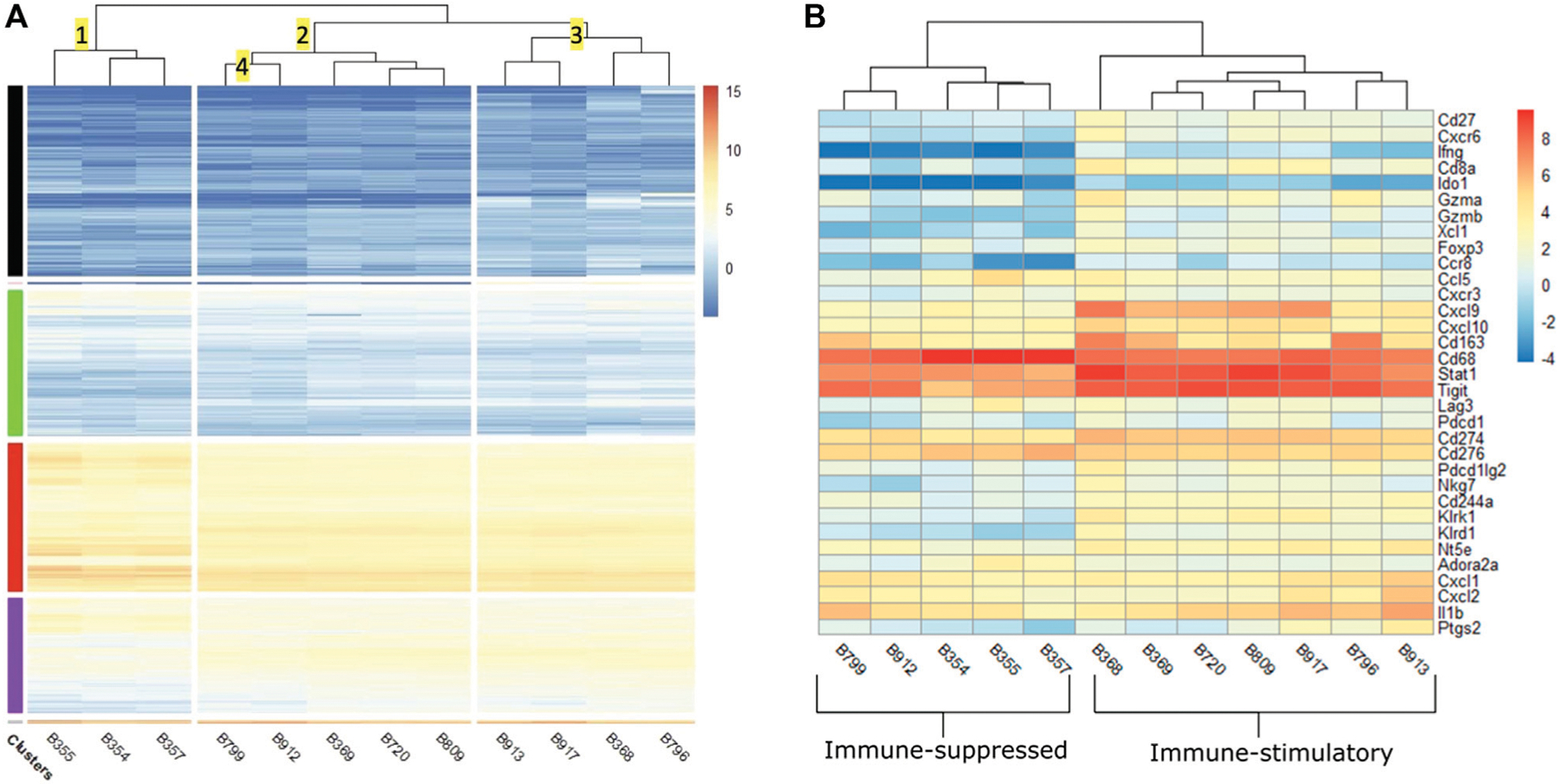
**(A)** Heatmap of log_2_ counts per million (logCPM) of the top 10,000 differentially expressed genes across 12 tumors. Samples (*x*-axis) are hierarchically clustered based on similarity of gene expression. The color scale indicates the intensity of intrinsic expression (logCPM). IST: immune-stimulatory, ISU: immune-suppressed, PBS: phosphate buffer saline. Tumors injected with saline classified as ISU are B355, B354, B357. Tumors received aPD1 gel classified as IST are B720, B917. aPD1/ADA gel treated tumors classified as IST are B369, B809, B913, B368, B796 and those classified as ISU are B799, B912. **(B)** Heatmap of logCPM of a subset of 33 immune and adenosine pathway signature genes. The color scale indicates the intensity of intrinsic expression (logCPM).

We first analyzed the RNA-Seq results by clustering the tumors based on the similarities of expression profiles. Figure 5A showed the heatmap of normalized expression levels (represented has log_2_ counts per million or logCPM) generated for the top 10,000 genes with the highest expression levels. Horizontal clusters (shown in colors of black, green, red and purple) represented unbiased clustering of genes with similar expression patterns. The hierarchical clustering (numbered) was established based on similar expression profiles across the samples. Cluster 1 contained saline-injected tumors (B355, B354, and B357) which were separated from other treated-tumors. This distinct separation suggested that treatments exerted a transcriptomic shift between treated and non-treated tumors. aPD1/ADA gel and aPD1 gel treated samples were widely distributed into clusters 2 and 3. This observation suggested that tumor heterogeneity existed within the same treatment group.

Cluster 2.1 contained B799 and B912 tumors which were treated with aPD1/ADA gel and designated as ISU. These two samples were distant from those that received the same treatment but demonstrated *in vivo* IST response (B913, B368, B796). This observation leveraged the impact of tumor transcriptomic polymorphism on *in vivo* response to aPD1 therapy. Despite the apparent divergent *in vivo* effects on T cells, both IST samples (B369, B720 and B809) and ISU samples (B799 and B912) were congregated in cluster 2. Cluster 2.3 were made up of B720 which was treated with aPD1 gel and B809 which was treated with aPD1/ADA gel. Similarly, in cluster 3.1, B917 treated with aPD1 gel was found next to B913 treated with aPD1/ADA gel. These observations indicate ADA did not impact the tumor transcriptome, or that its effects were overshadowed by the aPD1gel.

The dichotomous responses were not unexpected, as Yu *et al* have shown that Renca tumors exhibit phenotypic heterogeneity (Yu et al., 2018). In five Renca specimens that they examined, significant differential expressions were detected in multiple immune genes that include innate (CCL5, CXCL9, IL12a, IL17b), adaptive (CD8a, IFNɣ, GZMK) and metabolic (IDO1) pathways and mechanisms (Yu et al., 2018). Both cancerous and non-cancerous cells accumulateδ genetic heterogeneity over cell division and in response to environmental factors, including therapeutic agents. In our studies, each *in vivo* tumor passage likely drove unique genotypic and phenotypic changes. Our hierarchical clustering analyses revealed differences between both treated and nontreated tumors in addition to the transcriptomic heterogeneity in tumors that received the same treatment. A global distribution of the top 10,000 differentially expressed genes (DEGs) was also visualized in volcano plots (Supplementary Figure S5) and discussed in the Supplementary Data Sheet S1. We next elucidated the effects of the treatments by evaluating the pairwise comparisons of DEGs in a subset of immune-related genes.

#### Gene Set and Comparison

**FIGURE 6 |:**
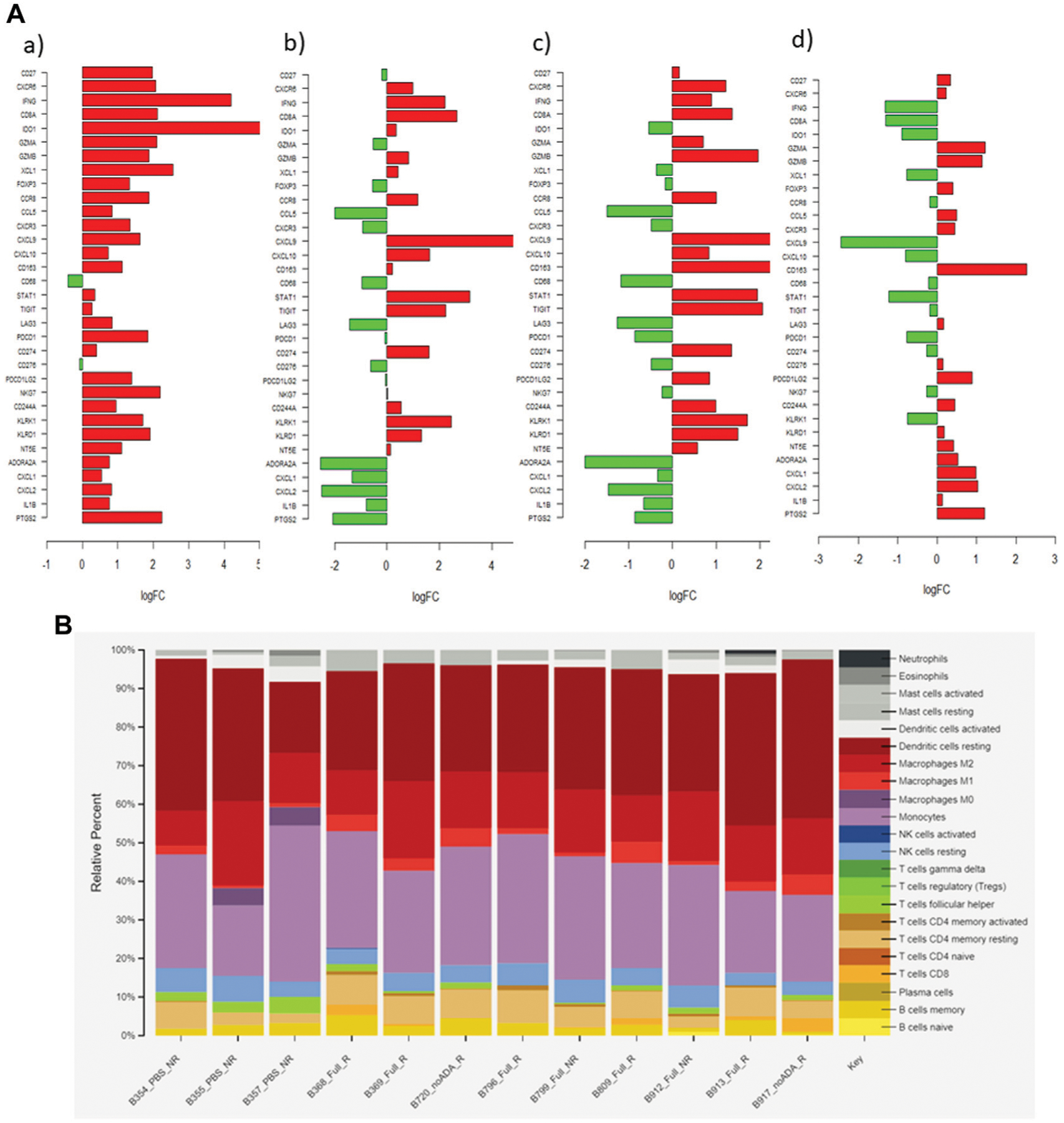
**(A)** Bar graph showing log_2_ fold-change (logFC) of 33 relevant genes in each pairwise comparison (a) ISU vs. IST (b) saline vs. aPD1/ADA gel (c) saline vs. aPD1 gel (d) aPD1 gel vs. aPD1/ADA gel. Positive logFC value (red) indicates an upregulation of gene in the latter group, while downregulation denotes the opposite. **(B)** CIBERSORTx and signature matrix LM22 delineation of immune cell subsets emerged from the samples’ responsiveness to the treatments.

The Quasi-likelihood F-test was used to evaluate DEGs between response and treatment groups. The null hypothesis is that all genes are expressed at the same level between the two groups. A log_2_FC ≥ 1 and an adjusted *p*-value of ≤0.05 was considered differentially regulated. The subset of genes selected for discussion in this study includes tumor-inflammation signature genes adopted from (Danaher et al., 2018), ADO-signature genes from (Fong et al., 2020), and other immune genes recognized as relevant in RCC (Supplementary Table S2; Figure 6). Because the ISU and IST tumors were classified based on *in vivo* results, this comparison was preliminarily employed to evaluate the relevance of these genes to the *in vivo* observations. In other words, an upregulation of a pro-inflammatory gene in the IST group would likely be correlated with a stimulatory *in vivo* response. Subsequently, an upregulation of genes in the treatment group might dictate the immune effect prompted by the treatment.

Prior to comparing the DEGs between IST and ISU, we reviewed the intrinsic expression of genes in all investigated tumors regardless of the response. The logCPM heatmap revealed high levels of CD63, TIGIT and STAT1 in both IST and ISU tumors (Figure 5B). Enrichment of these markers and mediators of activated macrophages, T cells and NK cells, indicates robust immune infiltration across all tumors. On the other end, high expression of genes facilitating VEGF signaling pathways, such as VEGFa, HIF1a, MYOF, MAPKAPK2 and NEDD4, existed in all tumors (Supplementary Figure S6). The presence of genes driving hypoxia and elevating interstitial pressure in the TME across the investigated Renca tumors supported the approach of retaining aPD1 and ADA with Z15_EAK gel. The expansion of both immune cells and pro-tumorigenic properties revealed a vigorous environment for treatment investigation.

The genes selected for IST-ISU validation included those that encode for immune cell markers, cytokines, and chemokines. The results showed upregulation of CD27 (3.9-fold) and CXCR6 (4.3-fold), T cell activation markers, in the IST tumors. An enrichment of the cytotoxic, pro-inflammatory markers IFNɣ (17.6-fold), CD8 alpha (4.4-fold), granzyme A (GZMA, 4.4-fold), and granzyme B (GZMB, 3.5-fold) was observed in the IST tumors. Notably, a low GZMB expression was recently reported to correlate with poor clinical outcomes in response to aPD1 therapy (Hurkmans et al., 2020). In addition, the IST tumors showed a 27.7-fold higher expression of IDO1, which was positively correlated with longer progression-free survival in patients with metastatic RCC sensitive to nivolumab (Seeber et al., 2018). The IST tumors also displayed a 1.8-fold increase in FOXP3, which was consistent with the qPCR results, likely an outcome of global T cell expansion, although the shift from FOXP3 toward IFNɣ phenotype indicates an activating phenotype. The induction of FOXP3 expression could be attributed to a specific property of tumor-associated macrophages (TAMs) in RCC (Eruslanov et al., 2013). A 3.5-fold upregulated expression of CCR8 on TAMs, detected in our immune-stimulatory samples, might have induced FoxP3 expressions on T cells via STAT3-mediated signaling (Eruslanov et al., 2013).

The IST tumors also exhibited distinct expression of markers for innate immune cells. Specifically, we detected upregulation of NK cell granule protein 7 (NKG7, 4.8-fold), killer cell lectin-like receptors (KLRK1, 2.9-fold and KLRD1, 3.6-fold), and chemokine ligand one gene (CXCL1, 6.6-fold). CXCL1 expression not only correlated with DCs accumulation but also enhanced CD8^+^ T cell activity in the TME of various mouse tumors (Böttcher et al., 2018). Additionally, the IST tumors showed an increase in CXCR3 (1.8-fold) and CXCL9 (2.7-fold), but not CXCL10. Pan *et al*. identified a similar expression pattern within Renca tumors in responding to IL-2 immunotherapy, which attenuated tumor growth in a CXCR3-dependent manner (Pan et al., 2006). CXCR3-dependent anti-tumor efficacy and upregulation of its ligands in mouse and human tumors were observed following PD-1/CTLA-4 checkpoint blockade (House et al., 2020). CXCR3, which is expressed on Th1, macrophage and NK cells, and its ligands (CXCL9, CXCL10) is an important chemokine axis in tumor suppression via interferon-induced cell-mediated immunity and inhibition of angiogenesis (Mantovani et al., 2004). A growing body of literature on the involvement of CXCR3 chemotaxis in RCC prognosis was recently summarized (Gudowska-Sawczuk et al., 2020). Our observation contributes to the current consensus that the upregulation of CXCR3 and its ligands correlates with Th1 responses and RCC human/murine tumor regression. In conjunction with Paul et al.’s finding, we speculate that an elevation of CXCL9 only (but not CXCL10) might be a characteristic of Renca tumors in response to immunotherapies.

#### Pairwise Comparison Across Treatments

After assembling the gene set from contrasting IST-ISU tumors, treatment-dependent DEGs were evaluated through pairwise comparisons of aPD1/ADA gel, saline and aPD1 gel (Supplementary Table S2; Figure 6). Tumors from mice that received aPD1/ADA gel demonstrated higher gene expressions of tumor suppressor components compared to those which received saline. Specifically, in aPD1/ADA gel samples we found upregulation of *Stat1* (3.8-fold), *Cd8a* (1.8-fold), *Gzmb* (3.8-fold), KLRK1 (2.9-fold), and *Klrd1* (2.2-fold). Less than one-fold change was detected in *Ifnγ*, *Gzma*, *Foxp3*, *Cd27*, *Cxcr6*, *Ido1* and *Nkg7*. No significant change was seen in *Cxcr3* or *Cxcl10*, but a higher *Cxcl9* was detected (6.1-fold). aPD1/ADA gel-treated tumors also demonstrated a 1.4-fold decrease in *Cd68* and 6.2-fold increase in *Cd163* compared to those treated with saline. M2 macrophages, identified by *Cd86* and *Cd163*, were associated with poor prognosis in patients with RCC (Kovaleva et al., 2016). The effects of aPD1 on TAMs should be investigated further.

Upregulation of *Cd274*, which encodes for PD-L1, was found in tumors received aPD1/ADA or aPD1 gel relative to saline. *Tigit*, which encodes the coinhibitory receptor on activated immune cells, was also upregulated compared to saline. *Pdcd1*, which encodes PD1 on T cells, however, was not affected by either treatment. Elevated TIGIT was found to correlate with PD1 expression on CD8^+^ TILs in various human and murine solid tumors (Johnston et al., 2014; Chauvin et al., 2015). The decoupled PD1 and TIGIT expressions could be a unique pathway in response to aPD1 therapy. In addition, *Ccl5* expression was reduced by aPD1/ADA gel and aPD1 gel (2.2-fold and 3.9-fold) in relative to saline. Elevated *Ccl5* expression was associated with cancer-related inflammation in RCC cell lines and poor clinical prognosis in patients with RCC (Gelbrich et al., 2017; Bai et al., 2020). Bai *et al* described pathways in which high *Ccl5* expression resulted in recruitment of tumor-infiltrating Tregs, which correlated with poor overall survival (Bai et al., 2020). *Ccl5*-deficiency mice had increased CD8^+^ T cells in tumors and reversal of aPD1 resistance in a colorectal cancer model (Zhang et al., 2018). Taken together with our observations, *Ccl5* may be a sensitive marker of aPD1 therapies in RCC.

DEGs between aPD1 gel and aPD1/ADA gel treated tumors were negligible, except that the presence of ADA significantly decreased the expression of *Cxcl9* (5.9-fold), which was not observed in other pairwise comparisons. Tumors treated with aPD1/ADA gel also exhibited a higher level of M2 macrophage marker CD163 (5.2-fold) compared to aPD1 gel. Among saline and aPD1 gel-treated tumors selected for analysis, *Cxcl9* (24-fold) and *Klrk1* (6.1-fold) were upregulated in the latter. Compared to saline, a 24-fold increase in *Cxcl9* by aPD1 gel versus a 6-fold increase by aPD1/ADA gel suggested that ADA reduced the extent of *Cxcl9* enrichment. While CXCL9 is classically associated with M1 macrophage (Mantovani et al., 2004), the chemokine also binds to tumor cells expressing CXCR3 receptor and exhibit pro-tumor effects (Neo and Lundqvist, 2020). Based on the pairwise comparisons, it can be concluded that *Cxcl9* is a sensitive marker for aPD1 and ADO-targeting combination strategy. Included in the analysis was a set of adenosine signature genes (*Cxcl1*, *Cxcl2*, *Cxcl3*, *Cxcl5*, *Cxcl6*, *Il8*, *Il1b*, *Ptgs2*), which were recently summarized based on RCC treatment-naïve patients (Fong et al., 2020). Most of these genes appeared to be absent or insignificant in our list of the 10,000 most differential expressed genes from our samples. Only *Cxcl1*, *Cxcl2*, *Il1b*, and *Ptgs2* exhibited logCPM values in the range of two–six. The ambiguous role of ADA in steering T cell responses could stem from that the tumors adapted to an ADO-independent phenotype *in situ*, during *in vivo* passage or in the mice used in testing the treatments. Finally, CIBERSORTx and signature matrix LM22 was used to delineate immune cell subsets emerged from the samples’ responsiveness to the treatments. The analysis shows a trend in which lymphocytes infiltration correlating with responding to aPD1/ADA gel injection (Figure 6B; Supplementary Material). Of the samples obtained from mice treated with aPD1/ADA gel, five of seven showed CD8 T cells, while the infiltration was found in one of three in samples of control saline injection. Additionally, Samples from all treatment groups showed the presence of activated CD4 T memory cells, while the subset was found in one of three tumors recovered from mice treated with saline. While these results were not necessarily significant, an immune activating trend of aPD1/ADA gel could be gleaned from the data.

## DISCUSSION

In the present study we investigated the impact of localized drug delivery targeting PD-1 and ADO, two immune checkpoints in the development of antitumor immunity. While locoregional immunotherapies moving toward mainstream (Marabelle et al., 2017; Meng et al., 2020), optimal therapeutic benefits are likely limited by poor retention of the biologics in tumors. Herein, we leveraged the self-assembling peptide Z15_EAK, which we discovered and charaterized to enhance local tissue retention (Pham et al., 2019). The formulations were delivered peritumorally instead of intratumorally to render sustained diffusion of antibodies to tumors and to target T cells traffficking to and from dLN. Jansen and others recently discovered in human kidney tumors a population of stem-cell-like T cells residing in the tumor niches near lymphatic vessels (Jansen et al., 2019). Using a clonotypic tracking system, the Chang group discovered that T cells responding to PD1 blockade are derived from a recently-infiltrated population rather than pre-existing exhausted T cell clones in the tumor (Yost et al., 2019). Another study shown that targeting immune checkpoints of T cells in dLN through locoregional delivery improved outcomes (Francis et al., 2020). These studies indicate that it may be more efficacous to target effector T cells in dLN rather than rescuing exhausted T cells in tumors. Thus, the results presented in the current study provide insights into dLN T cells modulation the context of locoregional delivery of IO agents.

To determine the effects of aPD1/ADA gel, we centered our analyses on profiling Tregs and effector T cells in tumors and dLN. Treatment with aPD1/ADA gel rendered delayed tumor growth relative to in mice treated with saline, aPD1/ADA solution, and aPD1 gel. In order to capture the diverging immune milieu at an early stage, our experimental design ended 2 days after the third dose and 12 days post inoculation. We postulated that the inhibitory effects of aPD1/ADA gel on tumor growth early in the process would enable tumor regression in the long term. Collective analysis of the functional and transcriptomic data indicates that treatment with aPD1/ADA gel reduced tolerogenic phenotypes and increased Th1 response compared to saline. Contrasting IST and ISU tumors revealed a set of genes that are differentially affected by the treatments in the model, altough the analysis was complicated by the inherent heterogeneity of the samples. Increasing the sample size and running the samples on a single flowcell would likely minimize the statistical noise. The contribution of ADA appeared to be limited in comparison to aPD1; it could be that the intervention should be timed during which high levels of ADO and effector T cells coincide (Sitkovsky, 2020).

Although the gel system was found to have limited impact in this model and mode of delivery, but the observations add to the current conversation in the feasibility and design of intratumoral and localized therapies using checkpoint inhibitors. The role of the gel might have been overshadowed by the therapeutic impact of aPD1 so future large scale studies would entail dose titration of aPD1. It would be ideal to compare all treatment groups in the same set of experiment. While the pairwise comparisons do not necessarily allow one to interpret effects of each permutation across all groups, they render direct evaluation of each component with reduced technical variations inherent in tumor growth kinetics. The approach is preventive in nature; we envision a logical translation of the concept would be to use the strategy to prevent cancer relapse after surgical resection. The gel would hold the therapeutics in place, modulating the local immune milieu. With this in mid, we analyzed the early local immune responses as a reflection of an active lesion rather than aiming for complete tumor regression and survival.

Ultimately, the data presented herein support the feasibility of localized delivery of immunotherapy in the context of RCC. The applications of this are manifold–many patients have locally advanced tumors that are not amenable to surgery; if these can be cytoreduced using locally applied therapy, it is possible that patients could be converted to surgical candidates. More broadly, the approach of local delivery holds the potential to mitigate side effects while still eliciting a systemic immune response. As immune-related adverse events remain a significant complication in systemic delivery of ICIs, local delivery is increasing becoming a viable alternative in selected patient populations.

## Supplementary Material

Supplemental

## Data Availability

The original contributions presented in the study are publicly available. This data can be found here: BioProject, PRJNA814277.

## Abbreviations:

ADA: Adenosine deaminase
aPD1: anti-PD1 antibody
aPD1/ADA gel: Z15_EAK self-assembling hydrogel mixed with anti-PD-1 IgG and ADA
aPD1 gel: Z15_EAK self-assembling hydrogel mixed with anti-PD-1 IgGIST Tumor samples classified as immune stimulatory based on relative increase in CD8 T cells and IFNg expression, and relative decrease in Tregs
ISU: Tumor samples classified as immune stimulatory based on relative decrease in CD8 T cells and IFNγ expression, and relative increase in Tregs
