# Supplementary material for: Localized PD-1 Blockade in a Mouse Model of Renal Cell Carcinoma
Source: Front Drug Deliv. Author manuscript; Available in PMC 2022 Sep 20. (PMC9486680; doi:10.3389/fddev.2022.838458)
Supplement: Supplemental [file NIHMS1833788-supplement-Supplemental.pdf]

Supplemental Method

Microfluidic antibody perfusion over Renca cells

A monolayer of Renca cells in complete medium were plated in µSlide channels and incubated for 4 hours. Saline or Z15_EAK solution was then introduced and incubated for 1 hour. Nonspecific antibody interactions were blocked by a perfusion of saline containing FBS. Fluorescein-labeled IgG was perfused over cells at a flow rate of 28.8µL/min, followed by a saline wash at 57.6µL/min to remove unbound antibodies. Images were acquired from identical x-y coordinates using the Olympus IX53 inverted microscope (Olympus, Shinjuku, Japan) at 20x magnification.

Tumor dissociation and preparation of single-cell suspensions

Renca primary in vivo tumor was processed mechanically/enzymatically following the protocol from the mouse Tumor Dissociation Kit (GentleMACS, Miltenyi Biotec #130-096-730). In brief, tumors were dissected into 2-4 mm fragments and placed in gentleMACS C tube containing 2.35 mL of RPMI 1640, 100 µL of Enzyme D, 50 µL of Enzyme R, and 12.5 µL of Enzyme A (provided in the kit). The mix was then subjected to two cycles of m_impTumor_02 program on gentleMACS Dissociator. After a 40-minute incubation at 37°C, another disaggregation run (m_impTumor_03) was performed before passing the tumor lysate through a 70 µm strainer. Tumor pellet was subjected to one red blood cell lysis cycle with ACK buffer while incubated on cold ice for 3 minutes. Processed tumor cells were counted with Countess II automated cell counter (ThermoFisher) and suspended in an appropriate volume of HBSS for in vivo injection.

RNA-sequencing and bioinformatics (detailed)

Extracted RNA were quantitated using the Qubit™ RNA BR Assay Kit (Thermo Fisher Scientific) followed by a RNA quality check using the Fragment Analyzer (AATI). For each sample, RNA libraries were prepared from 500ng RNA using the KAPA mRNA HyperPrep Kit (Kapa Biosystems) according to manufacturer's protocol, followed by a quality check using the Fragment Analyzer (AATI). Libraries with an average size of 350 bp (range: 200-600bp) were quantified by qPCR on the LightCycler 480 (Roche) using the KAPA qPCR quantification kit (KAPA biosystem). The libraries were normalized and pooled as per manufacturer protocol (Illumina). Sequencing was performed using NovaSeq 6000 platform (Illumina) to an average of 40M 101PE reads, on the NovaSeq SP-200 flowcell. Sequence quality was observed using FastQC using the default parameters and adapters sequences were removed

with TrimGalore [1, 2]. Paired-end reads were mapped to the mm10 reference genome using STAR [3]. Read quantification was performed using Subread's FeatureCount functionality specifying attribute type, feature type, countMultiMappingReads, countprimaryalignments, exon, and gene_id [4]. Before gene counts were normalized, low-expression genes were discarded using edgeR's [4] filterByExpr functionality. Gene counts were run through edgeR's calcNormFactors function, specifying the Trimmed Mean of M-values (TMM) algorithm as a means of normalization. Differential gene expression (DGE) analysis was performed with responder status, treatment, cohort, and sequencing batch listed as covariates in a generalized linear model evaluated by a quasi-likelihood F-test. P-Values were adjusted using the Benjamini–Hochberg procedure to control false discovery rate (FDR). Genes were considered differentially expressed if they met the following criteria: $|\log2| > 1$ and adjusted p-value < 0.05. Differentially expressed genes were analyzed via hierarchical clustering using the *pheatmap* package to visually assess variance amongst samples [5].

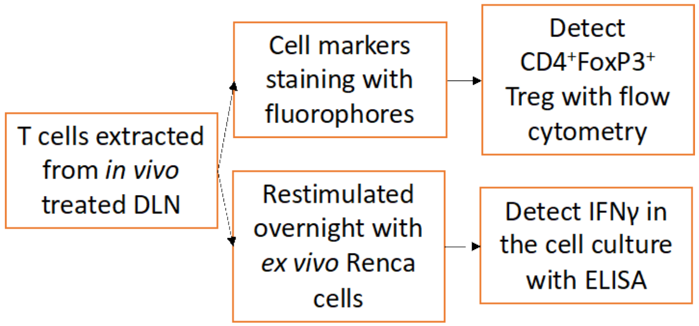

Design Matrix and Hierarchical Cluster Analysis

In total, 20076 genes were quantified after genes without sufficiently large counts were filtered out. The design matrix was set up using a generalized linear model to control for immune effect, treatment type, cohort, and batch effects, with groups being compared against the saline immune-suppressed control. The data was analyzed by controlling for the cohort and batch covariates in order to interrogate differences between pairwise treatment groups. This approach created four distinct analyses: immune suppressed vs immune stimulatory, saline vs aPD1/ADA gel, saline vs aPD1 gel, and aPD1 gel vs aPD1/ADA gel. To further examine how treatments affected the expression profile of the tumors, the top 10,000 genes with the highest expression levels were examined using hierarchical clustering.

Supplemental Results on RNAseq analyses

DEGs distribution

Volcano plots in Supplemental Figure 5 present the distribution of upregulation and downregulation in the top 10,000 genes with the highest expression level based on |FC| per comparison. The similarity between DEG distributions in Supplemental figures 5a and 5b indicates that aPD1 plays a larger role in altering tumor transcriptomes as opposed to ADA. While IST was compared to ISU in Supplemental Fig. 5c and aPD1/ADA gel was compared to aPD1 gel in Supplemental Fig. 5d, the similarities between DEG distributions may imply the role of ADA in the in vivo effects observed in IST samples. The spike in upregulated genes (in Supplemental Fig. 5c and 5d) contains predominantly genes encoded for keratin-associated proteins. It is noted that most IST tumors in the current analysis were treated with aPD1/ADA gel. Therefore, the similarity in gene distribution between Supplemental Fig. 5c and d is likely attributable to tumorigenic components such as keratin.

Supplemental Table 1 Six experiments (cohorts) in which Renca cells inoculated BALB/c mice received identical peritumoral dosing method and interval

Cohort ID	Saline (n)	aPD1/ADA gel (n)	aPD1 gel (n)	aPD1/ADA (n)
B3	5	5		
B5		5	5	
B6	5	5		
B7		5	5	
B8		5		5
B9	3	4	3	
Total	13	29	13	5

Supplemental Table 2: Differential expression of selected inflammatory and adenosine signature genes in Renca tumors

Upregulation in	**Immune-stimulatory**		**aPD1/ADA gel**		**aPD1/ADA gel**		**aPD1 gel**	
compare to	Immune-suppressed		saline		aPD1 gel		saline	
Gene	log_2FC	p-value	log_2FC	p-value	log_2FC	p-value	log_2FC	p-value
IFNG	4.2	0.0044	0.9	0.7438	-1.3	0.1241	2.2	0.4186
CD8A	2.1	0.0401	1.4	0.2693	-1.3	0.1576	2.7	0.1523
FOXP3	1.3	0.0304	-0.2	0.8156	0.4	0.4916	-0.6	0.5968
GZMA	2.1	0.0229	0.7	0.5069	1.2	0.1613	-0.5	0.7353
GZMB	1.9	0.0341	2.0	0.0920	1.1	0.1948	0.8	0.5974
NKG7	2.2	0.0090	-0.2	0.7817	-0.3	0.7095	0.0	0.9921
KLRK1	1.7	0.0074	1.7	0.0297	-0.7	0.1765	2.5	0.0345
KLRD1	1.9	0.0143	1.5	0.1143	0.2	0.7953	1.3	0.3196
CD244A	0.9	0.0301	1.0	0.0805	0.4	0.2833	0.5	0.4657
XCL1	2.6	0.0048	-0.4	0.6880	-0.8	0.2629	0.4	0.7634
CXCR3	1.3	0.0378	-0.5	0.5252	0.5	0.4557	-0.9	0.4009
CXCL9	1.6	0.0256	2.5	0.0143	-2.4	0.0047	4.9	0.0041
CXCL10	0.7	0.0897	0.8	0.1398	-0.8	0.0778	1.6	0.0625
CCR8	1.9	0.0256	1.0	0.3363	-0.2	0.8040	1.2	0.4226
CD68	-0.4	0.2414	-1.2	0.0271	-0.2	0.5436	-0.9	0.1724
CD163	1.1	0.0954	2.5	0.0136	2.3	0.0077	0.2	0.8692
STAT1	0.3	0.4253	1.9	0.0070	-1.2	0.0237	3.2	0.0053
CCL5	0.8	0.2061	-1.5	0.1083	0.5	0.4734	-2.0	0.1337
IDO1	5.3	0.0075	-0.5	0.4575	-0.9	0.3016	0.4	0.5789
CD27	2.0	0.0038	0.2	0.8276	0.3	0.5296	-0.2	0.8511
CXCR6	2.1	0.0186	1.2	0.2386	0.2	0.7721	1.0	0.4936
TIGIT	0.3	0.4386	2.1	0.0015	-0.2	0.6082	2.2	0.0086
LAG3	0.8	0.2448	-1.3	0.2037	0.2	0.8251	-1.4	0.3037
PDCD1	1.9	0.0164	-0.9	0.3258	-0.8	0.2457	-0.1	0.9408
CD274	0.4	0.1937	1.4	0.0059	-0.3	0.4148	1.6	0.0190
CD276	-0.1	0.8101	-0.5	0.2845	0.1	0.6741	-0.6	0.3308
PDCD1LG2	1.4	0.0561	0.8	0.3421	0.9	0.2234	0.0	0.9731

CXCL1	0.5	0.1677	-0.3	0.5048	1.0	0.0311	-1.3	0.0929
CXCL2	0.8	0.1916	-1.5	0.0943	1.0	0.1105	-2.5	0.0477
IL1b	0.8	0.2009	-0.7	0.4043	0.1	0.8287	-0.8	0.4782
PTGS2	2.2	0.0228	-0.9	0.4578	1.2	0.1582	-2.1	0.2041
NT5E	1.1	0.0136	0.6	0.2751	0.4	0.2966	0.1	0.8427
ADORA2A	0.8	0.1555	-2.0	0.0165	0.5	0.3368	-2.5	0.0241

*DEGs are obtained from transcriptome analysis of in vivo treated Renca tumors. A $\log_2$ fold change ≥ 1 suggests an upregulated DEG. A p-value ≤ 0.05 is considered to be statistically significant.

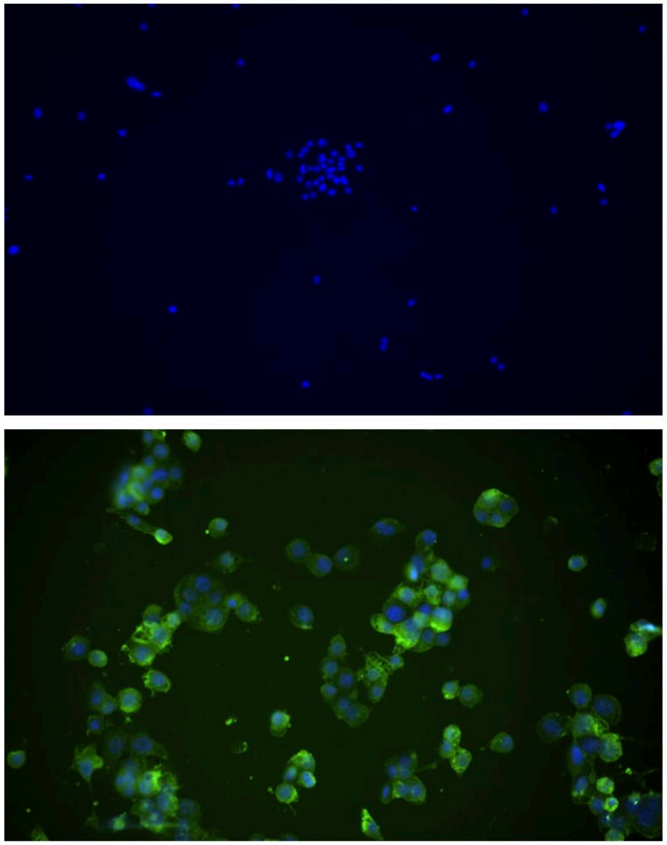

Supplemental Figure 1 Accumulation of antibodies on a monolayer of Renca cells in an µSlide channel microfluidic system. Saline (top image) or Z15_EAK solution (bottom image) were introduced and incubated with cells before a perfusion of FITC-labeled antibodies. Images acquired from the same location (79246X,39441Y) on the slide at 20X magnification (Olympus IX53 inverted microscope)

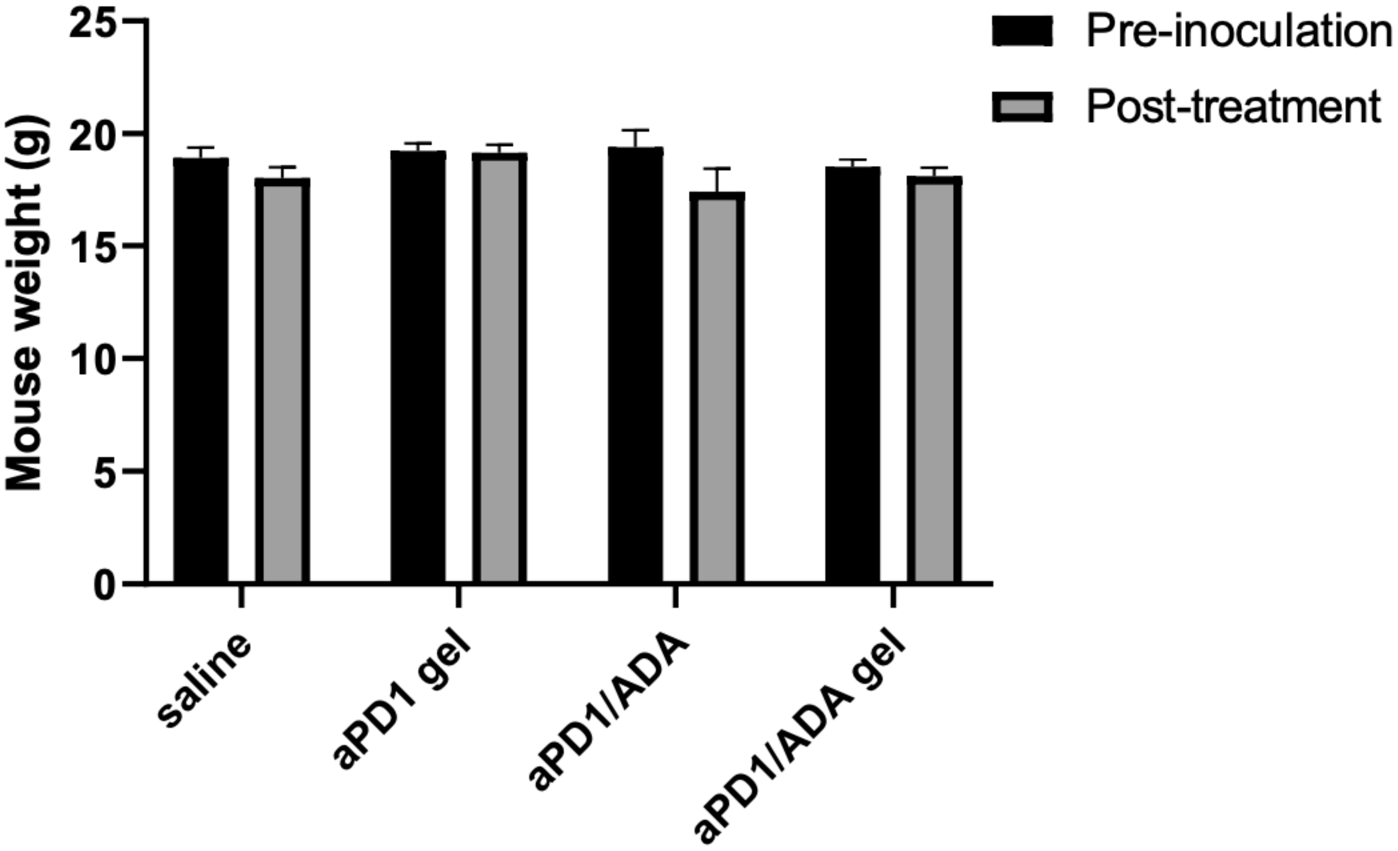

Supplemental Figure 2 Change in the body weight of Renca-tumor bearing mice receiving saline (n=13), aPD1 gel (n=13), aPD1/ADA (n=5) and aPD1/ADA gel (n=29) before tumor inoculation and at the end of experiment.

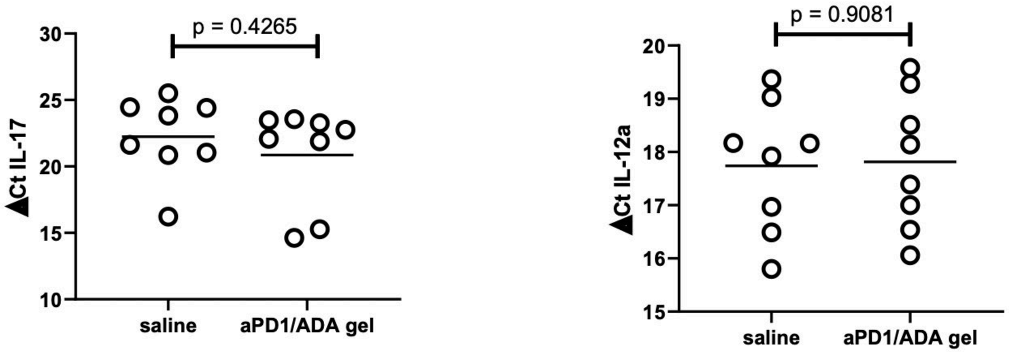
p = 0.4265
ΔCt IL-17
30
25
20
15
10
saline
aPD1/ADA gel
p = 0.9081
ΔCt IL-12a
20
19
18
17
16
15
saline
aPD1/ADA gel

(a)

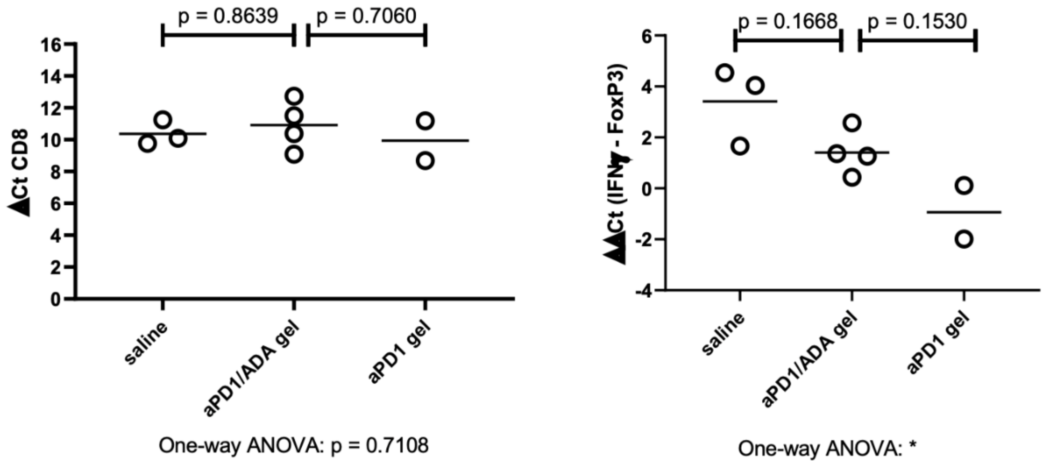
p = 0.8639
p = 0.7060
ΔCt CD8
16
14
12
10
8
6
4
2
0
saline
aPD1/ADA gel
aPD1 gel
One-way ANOVA: p = 0.7108
p = 0.1668
p = 0.1530
ΔΔCt (IFNγ - FoxP3)
6
4
2
0
-2
-4
saline
aPD1/ADA gel
aPD1 gel
One-way ANOVA: *

(b)

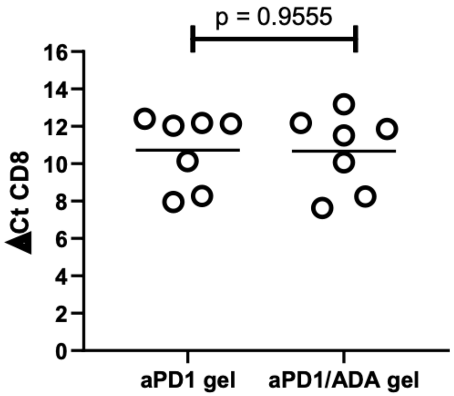

(c)

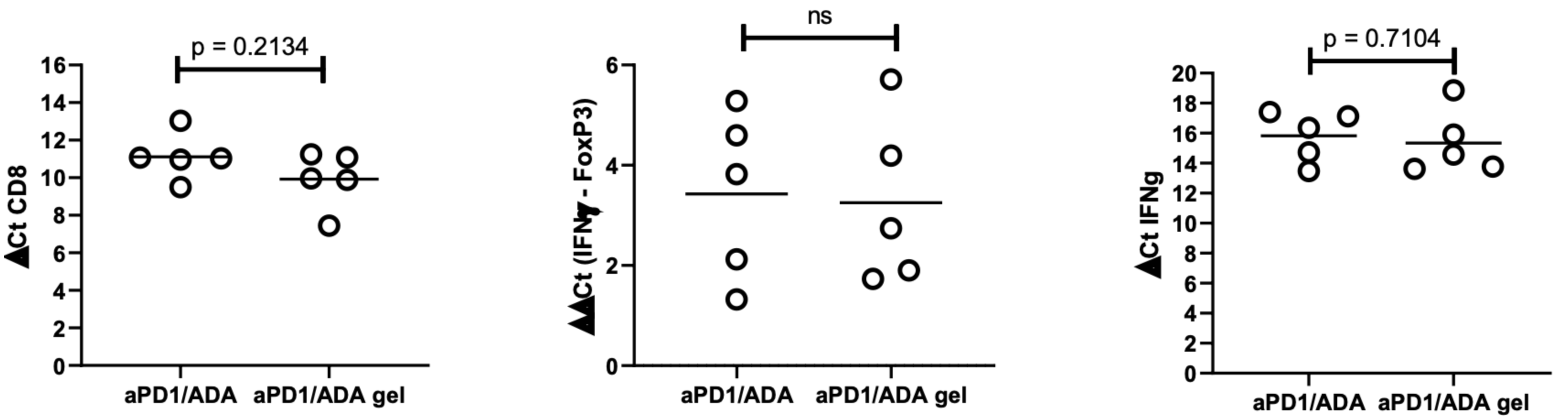

(d)

Supplemental Figure 3 (a) RT-qPCR of tumors extracted from two cohorts (B3 and B9) comparing aPD1/ADA gel and saline injection: insignificant difference in IL-17 and IL12a. (b) RT-qPCR results on CD8 and ddCt (IFNg-FoxP3) extracted from tumors in B9 cohort. (c) Insignificant difference of CD8 detected in aPD1/ADA gel and aPD1 gel treated tumors in cohorts B5 and B7. (d) RT-qPCR results on CD8, ddCt (IFNg-FoxP3), and IFNg extracted from tumors in B8 cohort. Two-tailed unpaired t-test or one-way ANOVA with multiple comparisons were employed for statistical analysis.

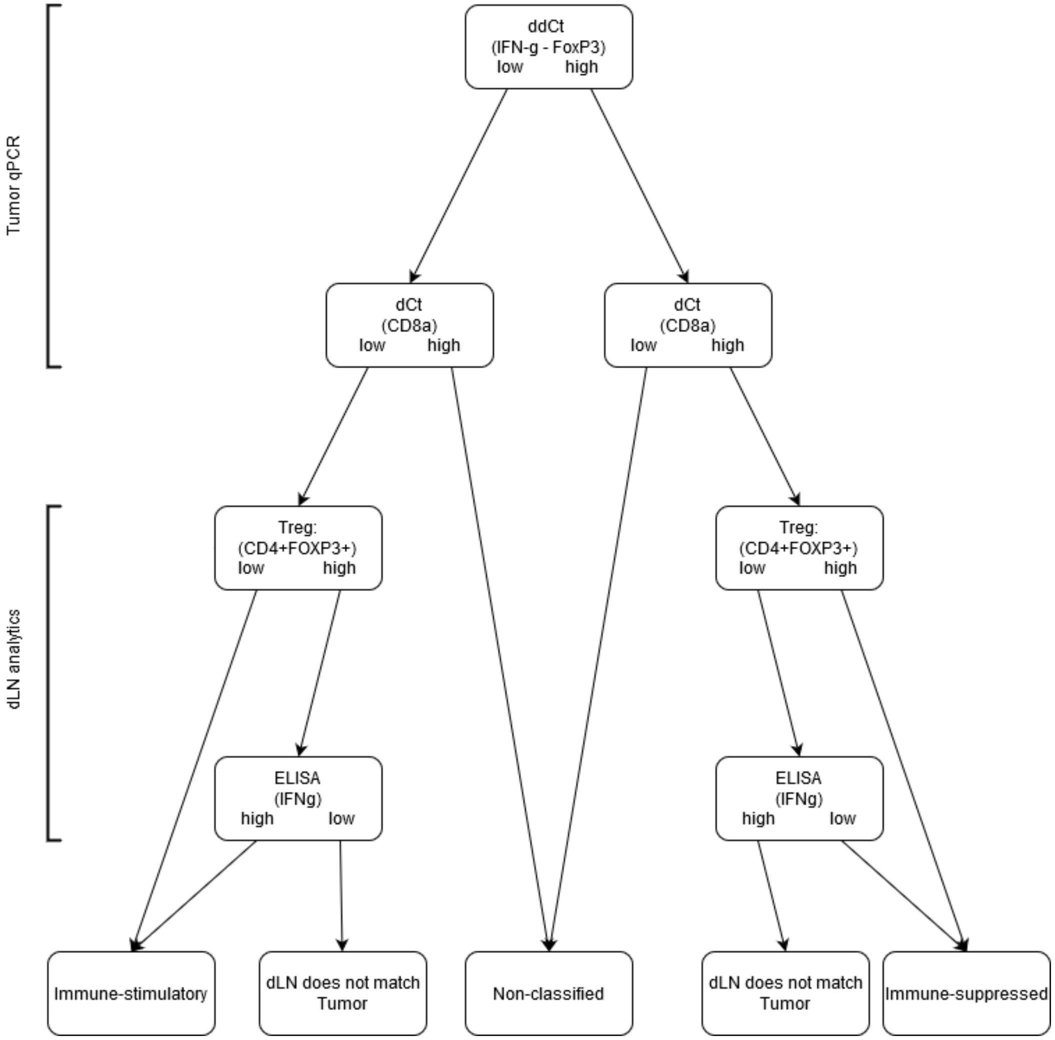

Supplemental Figure 4. Classification of immune-stimulatory (IST) and immune-suppressed (ISU) tumors based on the results of gene expressions (qPCR), dLN Treg frequency (flow cytometry) and IFNɣ production (ELISA) in dLN cell culture restimulated with RENCA cells. Samples were delineated according to the thresholds of ddCt (IFNɣ-FoxP3) <2 as low (or relatively high IFNɣ), dCt (CD8a) < 11 as low (high CD8 T cell infiltration), Tregs (CD4+FoxP3+%) significantly higher than the average frequencies in control groups, and IFNɣ higher than average concentrations in control groups. For example, mice with tumors and dLN classified as low

ddCt IFNɣ-FoxP3, low dCt CD8a, low Tregs % and high [IFNɣ] were classified as IST response. ISU mice are classified based on high ddCt (IFNɣ-FoxP3), high dCt CD8a, high Tregs % and low IFNɣ production in vitro. Not all samples simultaneously exhibited a consistent specification across four criteria, so a decision tree was made to illustrate the categorization process. Representatives from IST and ISU were selected for RNAseq. Mice that could not be classified were excluded.

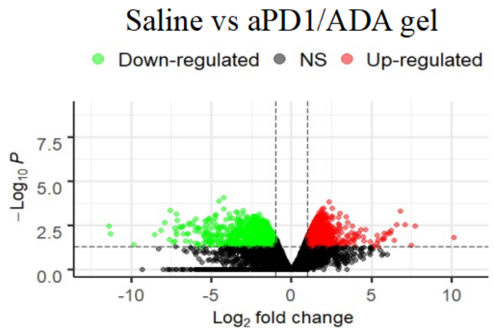

(a)

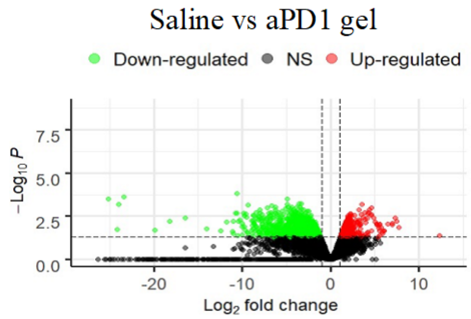

(b)

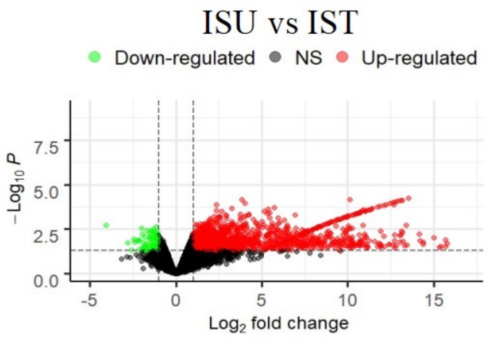

(c)

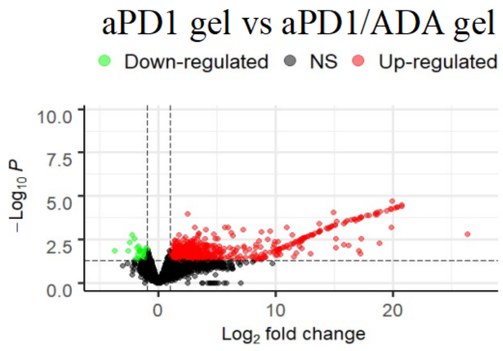

(d)

Supplemental Figure 5 Volcano plots of differential gene expressions per pairwise comparison (a) saline vs aPD1/ADA gel (b) saline vs aPD1 gel (c) IST vs ISU (d) aPD1 gel vs aPD1/ADA gel. Red denotes an upregulation of gene in the latter group following logFC > 1, p-value < 0.05 criteria. Genes were designated as downregulated (green) in the latter group if they met the following criteria:

logFC < 1, p-value < 0.05. Black denotes insignificant DEGs. P: p-value. Contrasting IST and ISU tumors shown 1644 DEGs, with 1566 upregulated and 78 downregulated. Contrasting saline and aPD1/ADA/gel tumors show 5627 DEGs, with 2518 upregulated and 3109 downregulated genes. aPD1 gel treated tumors demonstrated 3690 DEGs, with 1332 upregulated and 2358 downregulated genes compared to saline. aPD1 gel and aPD1/ADA gel comparison reveals 774 DEGs, with 743 upregulated and 31 downregulated genes in the latter group.

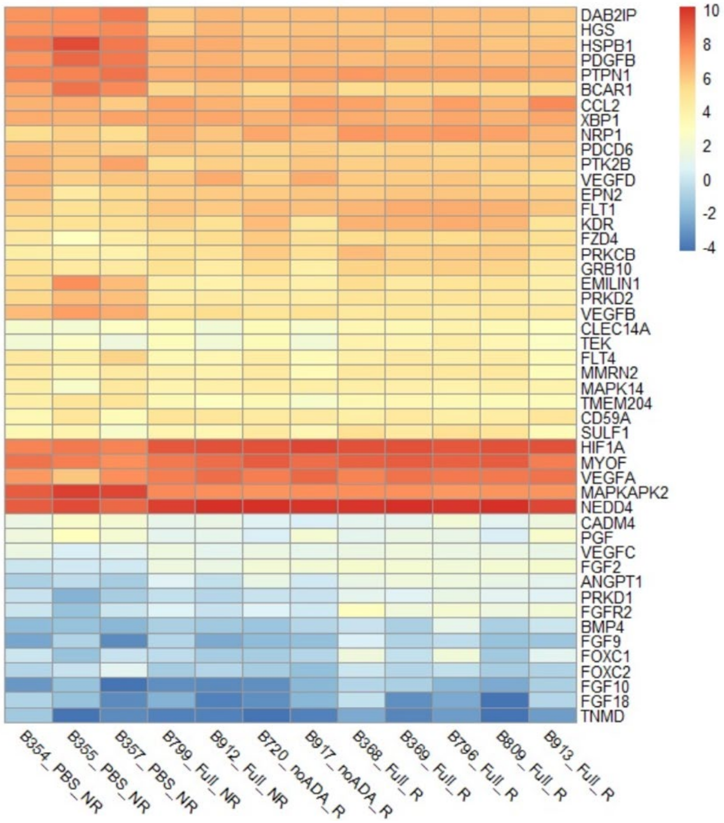

Supplemental Figure 6a Heatmaps of $\log_2$ counts per million (logCPM) across all the samples using 48 genes involved in Vascular endothelial growth factor (VEGF) receptor signaling pathway. Color scale reflects the value of logCPM. 46 genes were adopted from Mouse Genome Informatics Gene Oncology:0048010.

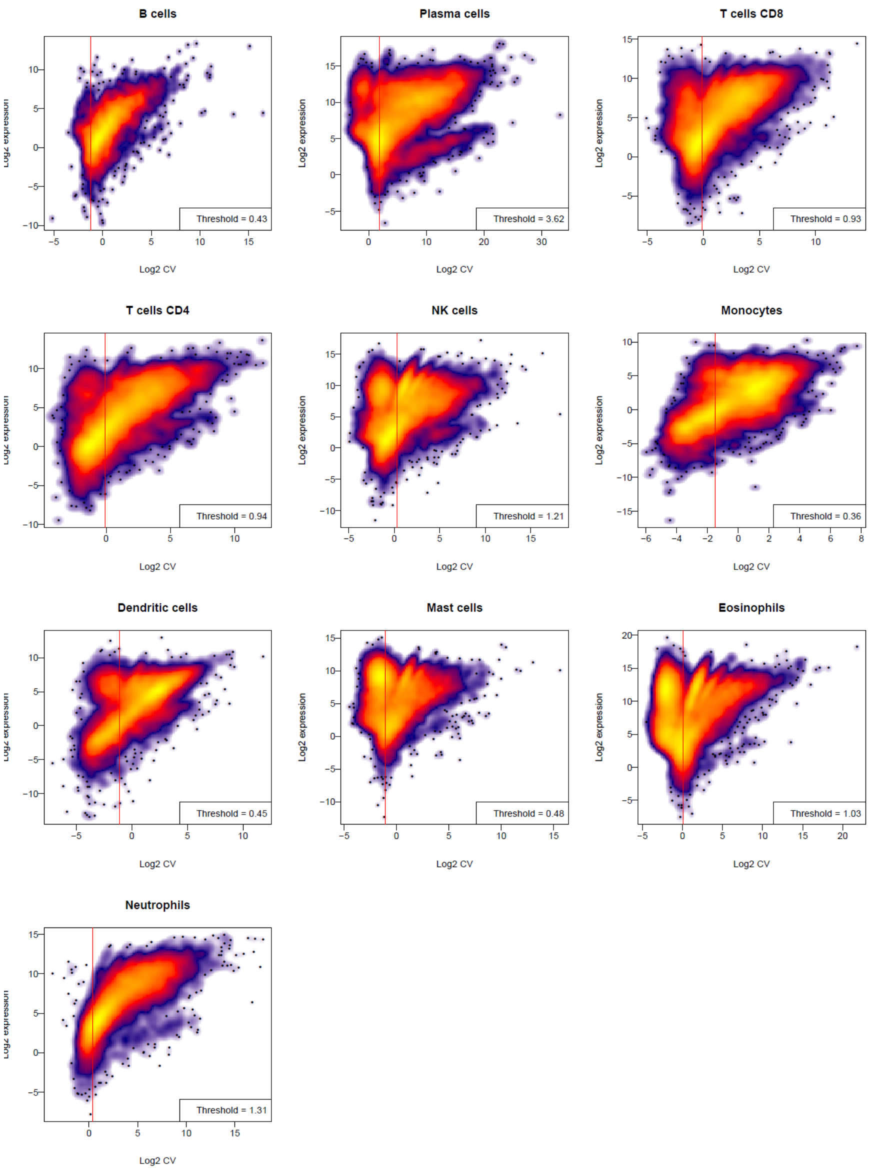

Supplemental Figure 6b Noise threshold generated from CIBERSORTx and signature matrix LM22.

1. Martin, M., *Cutadapt removes adapter sequences from high-throughput sequencing reads.* 2011, 2011. **17**(1): p. 3.
2. Andrews, S., *FastQC: a quality control tool for high throughput sequence data.* 2010, Babraham Bioinformatics.
3. Dobin, A., et al., *STAR: ultrafast universal RNA-seq aligner.* Bioinformatics, 2013. **29**(1): p. 15-21.
4. Liao, Y., G.K. Smyth, and W. Shi, *featureCounts: an efficient general purpose program for assigning sequence reads to genomic features.* Bioinformatics, 2014. **30**(7): p. 923-30.
5. Kolde, R., *Implementation of heatmaps that offers more control over dimensions and appearance.* 2015, CRAN.R Project.
